# Impulsivity relates to striatal gray matter volumes in humans: evidence from a delay discounting paradigm

**DOI:** 10.3389/fnhum.2015.00384

**Published:** 2015-07-02

**Authors:** Melanie Tschernegg, Belinda Pletzer, Philipp Schwartenbeck, Philipp Ludersdorfer, Uta Hoffmann, Martin Kronbichler

**Affiliations:** ^1^Centre for Cognitive Neuroscience, University of SalzburgSalzburg, Austria; ^2^Neuroscience Institute, Christian-Doppler-Klinik, Paracelsus Medical University SalzburgSalzburg, Austria

**Keywords:** MRI, delay discounting, striatum, impulsivity, gray matter volume

## Abstract

Time-stable personality traits, such as impulsivity and its relationship with functional and structural brain alterations, have gained much attention in the recent literature. Evidence from functional neuroimaging data implies an association between impulsivity and cortical as well as subcortical areas of the reward system. Discounting future rewards during impulsive decisions can be related to activation in the orbitofrontal cortex and striatum. Cortical structural changes in prefrontal regions have been found for introspective impulsivity measures. The present study focuses on brain regions associated with delay discounting to investigate structural manifestations of trait impulsivity. To test this, seventy subjects underwent structural magnetic resonance imaging (MRI) followed by a behavioral delay discounting task outside of the scanner to measure impulsivity with questions like: “Would you like to have 3€ immediately or 10€ in 5 days?”. The amount of smaller-but-sooner decisions was calculated and used as a measure of behavioral impulsivity. Furthermore, we estimated subject’s individual delay discounting parameter *K* reflecting the tendency to discount future rewards. Behaviorally, we found strong evidence in favor of a discounting utility model compared to a standard hyperbolic model of choice valuation. Neuronally, we focused on cortical and subcortical brain structure and investigated the association of behavioral impulsivity with delay discounting tendencies and gray matter volume. Voxel-based morphometric analyses showed positive correlations between delay discounting and gray matter volume in the striatum. Additional analyses using Freesurfer provided evidence for a positive correlation between delay discounting and gray matter volume of the caudate. Taken together, our study provides strong evidence for a structural manifestation of time-stable trait impulsivity in the human brain.

## Introduction

Impulsivity is a stable psychometric trait defined as “a predisposition toward rapid, unplanned reactions to internal or external stimuli without regard to the negative consequences of these reactions to the impulsive individual or to other” (Moeller et al., 2001, p. 1784). According to the DSM-IV (American Psychiatric Association, 2000) such reactions include: (i) blurting out answers before questions have been complete; (ii) difficulty awaiting turns; and (iii) interruption of others. Trait impulsivity is one of the major symptoms of attention deficit disorder and one of the major vulnerability factors for behavioral addiction (e.g., pathological gambling) and substance abuse (Dixon et al., 2006; Scheres and Sanfey, 2006; Businelle et al., 2010; Luman et al., 2010; Bickel et al., 2012). High impulsivity is associated with strong discounting of (negative) future consequences as well as an inability to wait for future rewards (for a review, see Green and Myerson, 2004).

A common task to measure individual impulsivity is the so-called delay-discounting (DD) task, in which subjects have to decide between a smaller-but-sooner (*SbS*) and a larger-but-later reward (e.g., “Would you like to have 3€ immediately or 10€ in 5 days?”). High impulsivity is associated with enhanced DD, such that impulsive individuals reliably prefer *SbS* over larger-but-later rewards (Simpson and Vuchinich, 2000; Pine et al., 2010; Dalley et al., 2011; Broos et al., 2012). The DD paradigm is an implicit measure of impulsivity. Such measures have one major advantage over (explicit) introspective questionnaires, such as the Barratt impulsiveness scale: they provide a direct assessment of the specific processes associated with impulsivity and are harder to manipulate than self-report scales (Reynolds et al., 2006).

In the neuroimaging literature, delay discounting has been associated with increased activation in brain regions involved in reward anticipation and outcome processing (Knutson et al., 2001), such as the striatum (caudate and putamen) and medial prefrontal regions, such as the orbitofrontal cortex and the anterior cingulate cortex (McClure et al., 2004; Hariri et al., 2006; Schmaal et al., 2012; for a review see Peters and Büchel, 2011). In particular, DD scores have been related to brain activation of the ventral striatum, such that high impulsivity has been associated with a “hyper-reactivity” of the ventral striatum signaling a preference for sooner rather than later rewards (McClure et al., 2004; Hariri et al., 2006). Contrary to that, inconsistencies in functional activation for delay discounting and reward related activation can be observed in hyper- as well as hypo-reactivity of reward circuits in functional data (for a review, see Leyton and Vezina, 2013; Limbrick-Oldfield et al., 2013).

Furthermore, resting state data suggest intrinsic functional manifestations of trait impulsivity in cortical as well as subcortical functional connectivity (Schmaal et al., 2012; Davis et al., 2013; Li et al., 2013). In particular, increased functional connectivity within the ventromedial prefrontal cortex and the striatum, posterior cingulate cortex, hippocampus as well as the parahippocampal area has been reported for high DD scores (Li et al., 2013). Moreover, Li et al. (2013) successfully predicted DD scores from resting-state functional connectivity networks. In addition, Schmaal et al. (2012) reported increased functional connectivity of the dorsal anterior cingulate cortex with midbrain regions like the ventral tegmental area and the substantia nigra for individuals with a high tendency for DD and suggested an association between task-free fMRI activation and impulsivity.

Results regarding structural differences associated with impulsivity are inconclusive. In healthy individuals, decreased gray-matter volumes of the orbitofrontal cortex have been related to high trait impulsivity in adults (Bjork et al., 2009; Matsuo et al., 2009) and adolescents (Schilling et al., 2013) using the Barratt impulsiveness scale, the Temperament and Character Inventory, as well as the delay discounting task respectively. Cho et al. (2013) found a positive relationship between impulsivity scores assessed with the Barratt impulsivity scale and medial prefrontal gray matter volumes. They also reported a negative relationship between impulsivity scores assessed with a 27-item delay discounting paradigm (Kirby et al., 1999) and gray matter volumes of the putamen bilaterally.

Of further interest are structural findings in patients with psychiatric conditions, which have been related to impulsivity, such as attention deficit hyperactivity disorder (ADHD; Nakao et al., 2011), suicidality in affective disorders (Dombrovski et al., 2012) substance abuse (Beck et al., 2009), behavioral addiction (Koehler et al., 2015) and psychopathy (Glenn et al., 2010). A meta-analysis of brain structural studies comparing ADHD patients of various ages to healthy controls found reduced striatal gray matter volumes due to ADHD (Nakao et al., 2011). Similarly, suicidality of patients with major depressive disorder was negatively related to gray matter volumes in the putamen, but positively to impulsivity assessed with a DD task (Dombrovski et al., 2012). Also, in substance abuse disorders, both decreased as well as increased gray matter volumes of the striatum have been reported (e.g., Potvin et al., 2007; Barrós-Loscertales et al., 2011). Consequently, a recent meta-analysis concludes that the most robust findings in substance abuse concern frontal areas with no effect on the striatum (Ersche et al., 2013). Research on addiction and affective disorders underpinned the importance of structural changes in subcortical reward system areas. On the other hand, behavioral addictions, such as pathological gambling, were associated with increased gray matter volumes in the striatum and pre-frontal cortex (Koehler et al., 2015). Similarly, highly impulsive, psychopathic individuals showed increased volumes of frontal areas and the striatum (Glenn et al., 2010).

To resolve these inconsistencies, the present study investigates the relationship between gray matter volume in cortical and subcortical regions implicated in reward processing and individual impulsivity traits assessed implicitly using a DD paradigm adopted from Pine et al. (2009) in healthy individuals. In order to get a more stable, behavioral measure of trait impulsivity, the present study included more items than the study of Cho et al. (2013) and DD scores were averaged over three different sessions to minimize the influence of state factors on assessing individual levels of impulsivity. A further advantage of the Pine et al. (2009) stimuli over the Kirby task (Kirby et al., 1999) is that participants both have to decide between later vs. immediate rewards and later vs. sooner (but not immediate) rewards. Pine et al. (2009, 2010) showed that the discounted utility model explains the behavioral data better than the standard hyperbolic model and we expect to replicate this result in a larger sample.

We used voxel-based morphometry (VBM) to assess the relation between DD and gray matter volumes. We additionally validated the VBM results with individually defined anatomical regions of interest as implemented in the Freesurfer software. This combination of group and individual analyses in a large sample provides detailed insight in the localization of structural correlates of impulsivity.

Several neuroimaging studies have already reported higher activation in the striatum, particularly the caudate nucleus and putamen, in participants with high DD scores (McClure et al., 2004; Hariri et al., 2006; Pine et al., 2010; Davis et al., 2013; Li et al., 2013; for reviews, see Blum et al., 2000; Cardinal, 2006; Dalley et al., 2011), but there is still not much evidence whether these functional changes are accompanied by structural changes of the striatum.

Given the fact that cortical as well as subcortical regions show stronger functional activation in trait impulsivity and related pathological conditions, we expect to find a positive relationship between striatal regions, particularly the caudate, gray matter volumes and delay discounting scores.

## Materials and Methods

### Subjects and Procedure

Fifty-one women (mean age = 24.50 years; SD = 5.3; range = 19–43 years) and 19 men (mean age = 27.15 years; SD = 7.22, range 20–44 years) participated in this study. Of the 53 female participants, 20 were naturally cycling, 32 were users of oral hormonal contraceptives. According to self-reports, all participants were healthy (no psychiatric or neurological illness) and not currently on medication. Participants did not show any brain tissue abnormalities on the structural magnetic resonance imaging (MRI).

All subjects gave written informed consent to participate in the study. All methods conformed to the Code of Ethics of the World Medical Association (Declaration of Helsinki). The institutional guidelines of the University of Salzburg (Statutes of the University of Salzburg—see https://online.uni-salzburg.at/plus_online/wbMitteilungsblaetter.display?pNr=98160) state in § 163 (1) that ethical approval is necessary for research on human subjects if it affects the physical or psychological integrity, the right for privacy or other important rights or interests of the subjects or their dependents. In § 163 (2) it is stated that it is the responsibility of the PI to decide, whether § (1) applies to a study or not. Therefore we did not approach our institutional review board to obtain ethical approval or a waiver for this study. Since it was non-invasive and performed on healthy adult volunteers who gave their informed consent to participate, § (1) did not apply. Participants responded voluntarily to recruitment ads posted at bulletin boards at our institution. Data was recorded in anonymized/deidentified form and saved on a password-protected server. Upon arrival at the lab, participants were assigned a subject ID (v001, v002, etc.), which was used throughout the study.

### Behavioral Assessment

After the scanning session, participants completed a computerized DD task three times within 1 month to provide more realistic estimates of trait impulsivity by minimizing state influences. Participants performed a modified computer version of a DD task adapted from Pine et al. (2009). Participants had to choose between *SbS* (with a maximum time delay of 2 weeks) and larger-but-later rewards (with a maximum time delay of 1 year), for example: “Would you prefer 5€ in 2 weeks or 15€ in 7 months?” Both options (smaller and larger rewards) were presented simultaneously on the computer screen and participants were instructed to indicate their choice using the left or right mouse button, respectively. Participants were not paid for participation but were instructed to make realistic choices. Several studies have shown that participant’s choices do not differ between hypothetical and real outcomes (Johnson and Bickel, 2002; Lagorio and Madden, 2005).

Each of the three sessions consisted of 110 trials. Ten of these trials, were control trials were the same options were presented on the left and right side of the screen to control for possible left or right hand response bias. In the remaining 100 trials, *SbS* and larger-but-later options were randomly presented on the left and right side of the screen. Reward value and delay duration/time were randomized across trials, as well as across the three sessions. The averaged reward value and delay time for *SbS* and larger-but-later options was matched across the three stimulus sets, which were counterbalanced across sessions.

#### DD Parameter

We averaged the number of *SbS* decisions over the three sessions. These values are simple behavioral measures reflecting the relative frequency of how often the *SbS* option was preferred over the larger-but-later option. The *SbS* scores reflect the devaluation of delays and thus reflect behavioral impulsivity in such choices.

*K* additionally represents the individual tendency for discounting future rewards in relation to the presented alternative. Thus, individuals with high *K* display stronger DD and show an elevated preference for sooner rewards. In Pine et al. (2009) the DD parameter *K* is estimated using a so-called discounted utility model, which outperformed a standard hyperbolic model in explaining the behavioral data. In the present study we also compared the performance of both models and estimated *K* according to the winning one.

Briefly, in the discounted utility model the value of an option is determined by

(1) $$V=\frac{1-e^{\left(-rM\right)}}{r(1+K\;\cdot \;d)}$$

Here, *M* refers to the magnitude or amount of the monetary offer and *d* to the temporal delay. The parameter *K* determines a subject’s tendency to discount rewards in the future and thus reflects a subject’s impulsivity or tendency to choose *SbS* options. In contrast, *r* refers to the curvature of the utility function, such that the function is concave for positive and convex for negative values of *r*.

Often, the discounted utility model is compared to a simpler, standard hyperbolic model, where the value of an option is determined by

(2) $$V=\frac{M}{\left(1+K\;\cdot \;d\right)}$$

Note that in the standard hyperbolic model there is no *r*-parameter referring to the curvature of the relationship. Both models rely on a softmax function for producing choice probabilities based on the values of the options including an inverse temperature (beta) parameter that determines the stochasticity of choice (see Pine et al., 2009, 2010 for details).

We used both models and estimated the free parameters based on observed behavior using maximum-likelihood estimation. Free parameters of the discounted utility model are *r*, *K* and beta whereas in the hyperbolic model only *K* and beta are estimated. Finally, we performed random effects Bayesian model comparison to determine which of the two models accounted better for observed behavior.

### MRI Data Acquisition

T1-weighted gradient echo pulse sequences (turbo field echo) were acquired by a 3 T Siemens TIM TRIO magnetic resonance imaging scanner with a 32-channel head coil and the following scan parameters: TR: 2,300 ms; TE: 2.91 ms; TI: 900 ms; flip angle: 9°; FOV: 256 × 256; 160 slices; voxel size: 1 × 1.2 × 1 mm.

### Voxel Based Morphometry (VBM)

Analysis of structural data was conducted with the VBM 8 toolbox^1^ of the SPM 8 software^2^. High resolution T1-weighted images were preprocessed within the following steps in the VBM 8 toolbox: (1) correction of the intrasubject bias using spatial normalization to the same stereotactic space; (2) segmentation of the gray and white matter; (3) linear (affine) and nonlinear registration using DARTEL; and (4) modulation of the different brain segments using nonlinear normalization parameter to control for individual differences in brain size. We refined segmentation with a Markov Random Field Model (Cuadra et al., 2005) using posteriori rating (Rajapakse et al., 1997) to get partial volume effects (Tohka et al., 2004). Modulation by nonlinear effects ensured the correction for differences in brain size. Smoothing was conducted using a 8 mm full width at half maximum (FWHM) Gaussian kernel.

### Freesurfer Analyses

For additional validation of the VBM analyses, gray matter volume of following subcortical seed regions was extracted with Freesurfer v.5.0^3^: caudate, putamen, pallidum, hippocampus, amygdala, and nucleus accumbens. Freesurfer (Athinoula A. Martinos Center for Biomedical Imaging, Harvard University, Cambridge, MA, USA) provides a fully automatic segmentation procedure of cortical and subcortical brain structures. A substantial advantage of this procedure over VBM is that individual brains are not normalized to a standard space. This results in a precise definition of brain regions based on the individual anatomy. For a quantitative assessment of subcortical brain structure automatic non-biased atlas based Bayesian segmentation was performed.

Standardized steps for preprocessing within Freesurfer were accomplished in the following order: movement correction, extracting of brain structure, automatic spatial transformation and segmentation of white matter in subcortical volume structures, intensity normalization, gray-white matter tessellation and topological structure. The Freesurfer data were checked visually after preprocessing. Gray matter volumes of subcortical regions were extracted by using Anglophone Parents’ Association in the Region of Chantilly (APARC) atlas (2005) in Freesurfer, including six subcortical structures on each hemisphere. To control for individual brain size as a possible covariate, additionally whole brain volume was extracted.

### Statistics

For the VBM analyses, the impulsivity parameter *SbS* and *K* were regressed voxel-wise on gray matter volumes in SPM8. Statistical analyses on the extracted regional gray matter volumes (Freesurfer) were done using PASW 20 (IBM SPSS Statistics, Chicago, IL, USA). The impulsivity parameters *SbS* and *K* were correlated with each subcortical region described above, while whole brain volume was controlled. We conducted 24 statistical tests in SPSS, because six subcortical regions on each hemisphere were correlated with the DD parameters. To control for multiple comparisons a Bonferroni correction was used, we thus adjusted the critical *p*-value to *p* = 0.002.

Impulsivity has been found to decline with age (Steinberg et al., 2008), as well as different impulsivity characteristics reported between men and women (Chamorro et al., 2012). Evidences suggest gray matter reductions in the bilateral caudate, pallidum, amygdala and hippocampus over lifespan (Giorgio et al., 2010). Furthermore, there are evidences for sex-specific structural brain differences (Giedd et al., 2012). The gray matter reduction with age in the caudate was furthermore found to be sex-specific, i.e., was confirmed in women, but not in men (Koikkalainen et al., 2007). We consider our sample as representative in terms of age and hormonal status. Nevertheless, in all analyses we controlled for gender and age in a second step to assess their possible moderating effects on the relationship between the parameter *K* and volume of subcortical structures.

## Results

### Behavioral Data

#### Model Comparison

**Figure 1:**
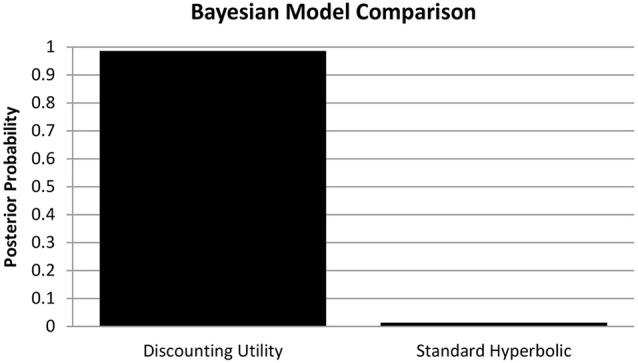
**Bayesian Model Comparison**. Posterior model probabilities of the random-effects Bayesian model comparison, providing evidence for the discounting utility model (*p* = 0.9864) over a standard hyperbolic model (*p* = 0.0136) of choice valuation in accordance with Pine et al. (2009).

In analogy to Pine et al. (2009) we computed the individual Akaike Information Criterion (AIC) scores as a measure of goodness-of-fit for the discounting utility and standard hyperbolic model. We then performed random effects Bayesian model comparison (see Figure 1) based on these values (Stephan et al., 2009) and found substantial evidence in favor of the discounted utility model (*φ* > 0.99, *sum*_discount_ = 17856.39 *sum*_hyperbolic_ = 18699.72). Therefore, we used the estimated parameters from the discounted utility model in our further analyses (see Table 1 for descriptive statistics of all estimated parameters).

**Table 1:** **Descriptive statistics of the delay discounting (DD) parameters**.

Parameter	Mean	Median	SD
*Discounted utility model*
*K*	1.54	0.03	7.56
*r*	0.03	0.01	0.10
*Beta*	7.04	3.64	8.28
*Standard hyperbolic model*
*Beta*	7.04	3.64	8.28
*K*	7.06	0.07	17.28
*Smaller but sooner decisions*	38.17	44.17	19.08

#### DD Parameters

Since we detected no significant deviation from a normal distribution for the amount of *SbS* decisions (*Z* = 1.190; *p* = 118), the mean of three sessions was taken for further behavioral and neuroimaging analysis (see Table 1 for descriptive statistics). There was no difference between men and women in the amount of *SbS* decisions (*t* = 1.502; *p* = 0.138). Furthermore, *SbS* decisions were not significantly correlated with age (*r* = −0.097; n.s.).

A Kolmogorov-Smirnov-Test was conducted to test for the normal distribution of *K*-values from the discounting utility model, which was significant (*Z* = 3.592; *p* < 0.001), thus we log-transformed the mean of the *K*-value for all further parametric tests (see Table 1 for the descriptive statistics of the actual *K*-values). *K* was not significantly correlated with age (*r* = 105; n.s.) and furthermore, there was no difference between men and women for the parameter *K* (*t* = −0.732; n.s.). The DD parameter *SbS* and *K* were significantly correlated (*r* = 0.438; *p* < 0.001).

### Neuroimaging Data

#### VBM Analyses

Voxel-wise whole brain correlations with *SbS* revealed two clusters (statistical threshold: *p* < 0.05 voxel wise with an additional cluster extent threshold of *p* < 0.05, FWE corrected). In the left hemisphere, a cluster at Montreal Neurological Institute (MNI) coordinates [−9 0 13] was identified that consisted of 1299 voxels (peak *t* = 3.85). In the right hemisphere a similar cluster at [9 6 16] was identified consisting of 2038 voxels (peak *t* = 5.05).

In both voxel-wise whole brain correlations the clusters comprise the bilateral caudate and the putamen [Harvard Oxford Subcortical Structural Atlas (as implemented in FSL, thresholded at 25%)]^4^.

**Figure 2:**
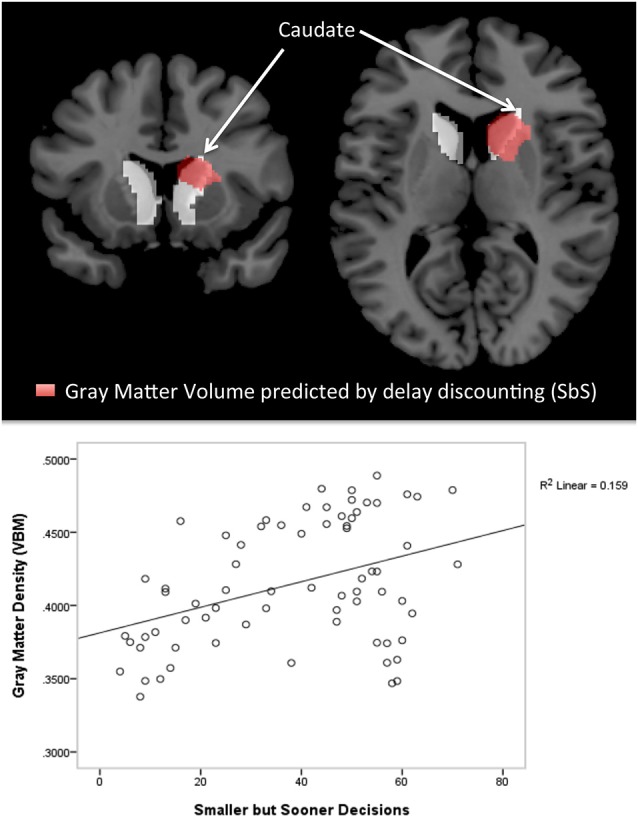
**Voxel-based morphometry (VBM) Results**. The top panel shows cluster with significant correlations between the delay discounting parameter *SbS* and gray matter volumes (controlled for gender and age) in the voxel-wise analysis (in red), superimposed by the anatomical mask for the caudate (in white); 1507 voxel in the right hemisphere, peak at [9 8 12]; According to the Harvard-Oxford Subcortical Structural Atlas [as implemented in FSL, http://www.fmrib.ox.ac.uk/fsl) thresholded at 25%] this cluster correspond to the right caudate (*p* < 0.001 cluster level FWE correction, *p* < 0.05 peak level FWE correction). The bottom panel shows the scatterplot of the correlation between gray matter density (VBM) and delay discounting.

Figure 2 shows the significant cluster for voxel-wise whole brain correlations with *SbS* decisions, controlled for age and gender. After controlling for gender and age, a similar cluster emerged consisting of 1507 voxels on the right hemisphere (at [9 8 12], peak *t* = 4.70, with an additional cluster extent threshold of *p* < 0.05, FWE corrected). According to the Harvard Oxford Subcortical Structural Atlas, this cluster compromises the right caudate. As can be seen in the lower panel of Figure 2, outliers did clearly not drive the relation between gray matter volumes and *SbS* decisions.

Voxel-wise whole brain correlation with *K*-values estimated from the discounting utility model exposed one significant cluster in the right hemisphere (at [15 27 −9], peak *t* = 7.06, *p* < 0.05, FWE corrected), compromising 37 voxel. After controlling for age and gender, voxel-wise whole brain correlation with *K*-values exposed the same cluster (*p* > 0.05, FWE corrected) in the right hemisphere (33 voxel at MNI coordinates [15 27 −9]). The cluster compromises the right caudate (at the peak [18 26 −6] and extents to the frontal lobe (Harvard Oxford Subcortical Structural Atlas, Harvard Oxford Cortical Structural Atlas).

#### Freesurfer Analyses—Subcortical Regions

**Figure 3:**
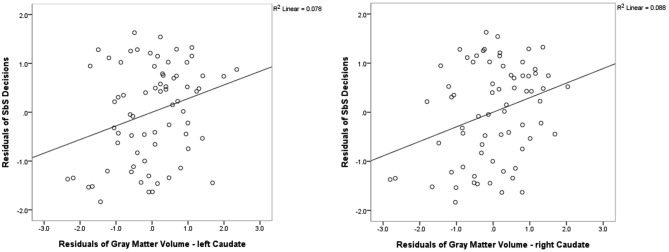
**Freesurfer Results**. Partial correlations between the bilateral caudate and delay discounting parameter *SbS* by displaying the residuals of *SbS* (controlled for whole gray matter cortex volume, gender and age) on the y-axis and residuals of caudate gray-matter volume (controlled for whole gray matter cortex volume, gender and age) on the *x*-axis.

We found positive correlations between the DD parameter *SbS* and volume (controlled for whole cortex volume, gender and age) in the left caudate (*r* = 0.284; *p* = 0.020, uncorrected) and in the right caudate (*r* = 0.313, *p* = 0.010, uncorrected). Correlations with no other subcortical region reached significance (all |*r*| < 0.322, all *p* > 0.864). See Figure 3 for the scatterplots of the correlations between the residuals of *SbS* and the left as well as the right caudate, controlled for whole brain volume, gender and age.

There was no significant correlation between *K*-values and cortical regions (all |*r*| < 0.455, all *p* > 0.522).

## Discussion

In this study, we obtained structural scans and behavioral measures from a DD task in 70 subjects. In accordance with results from Pine et al. (2009, 2010), we found substantial evidence for a discounting utility model of choice valuation over a standard hyperbolic model. Neuronally, we found that the amount of *SbS* decisions as well as the estimated *K*-parameters from the winning model, which reflect behavioral impulsivity as well as individual tendencies to devalue future reward, correlated with striatal gray matter volume.

Functional differences in individuals with high and low impulsivity have been demonstrated for frontal and striatal regions (McClure et al., 2004; Pine et al., 2009; Peters and Büchel, 2011). Furthermore, structural differences related to impulsivity have been reported in the orbitofrontal cortex (Bjork et al., 2009; Matsuo et al., 2009; Schilling et al., 2012). However, reports on gray matter changes reflecting high impulsivity in the striatum have been inconsistent (Glenn et al., 2010; Cho et al., 2013).

The present study provides strong evidence for a positive correlation between striatal gray matter volumes and trait impulsivity, as measured with the DD task by Pine et al. (2009). We show that trait impulsivity was related to gray matter volumes in the bilateral caudate and pallidum, such that gray matter volume was higher in individuals with high DD scores. This suggests, that a hyper-activity in mesolimbic areas (McClure et al., 2004; Beck et al., 2009), as well as connectivity changes in intrinsic fronto-striatal functional networks (Schmaal et al., 2012; Davis et al., 2013; Li et al., 2013) in highly impulsive individuals are accompanied by outlasting structural changes in the striatum.

Two important differences between the results of the present study and the previous study by Cho et al. (2013) were observed. First, our findings concerned the caudate and putamen, whereas in the study of Cho et al. (2013) the relationship between DD and striatal gray matter volumes was restricted to the putamen. Second, we found a positive relationship between DD and gray matter volumes in the striatum, while Cho et al. (2013) reported a negative relationship. The present findings are consistent with both previous functional imaging studies (McClure et al., 2004; Beck et al., 2009; Pine et al., 2009) as well as findings in psychopathic individuals (Glenn et al., 2010). The present study obtained a more comprehensive measure of DD as more items were included, testing was repeated in three sessions and discounting was assessed both in comparison to immediate and sooner (but not immediate) rewards.

Interestingly, in the present study we did not find a relationship between impulsivity and gray matter volumes in frontal areas, which is contrary to previous studies relating trait impulsivity to orbitofrontal gray matter volumes (Matsuo et al., 2009; Schilling et al., 2012). Even at a very liberal threshold (*p* < 0.01 uncorrected) we found no association between structural differences in prefrontal regions and DD measures. One possible explanation for this divergence might be the use of an implicit behavioral measure of impulsivity, i.e., the DD scores, as opposed to self-report questionnaires that were employed in previous studies (Matsuo et al., 2009; Schilling et al., 2012). Several studies suggest that the results obtained by self-report questionnaires may not adequately reflect objective or implicit measures of personality traits (e.g., Reynolds et al., 2006). Consequently, we speculate that subcortical regions may play a stronger role for the implicit behavioral aspects of impulsivity, such as reward representation, whereas frontal regions may be more strongly involved in the explicit self-perception of impulsivity or the (cognitive) control of impulsive behaviors (Knutson et al., 2001).

This idea is in line with findings showing structural alterations in the striatum in mental disorders associated with impulsivity (Ersche et al., 2011). There are still inconsistent findings with mental disorders like depression or addiction. Several studies found decreased gray matter volume in the orbitofrontal cortex, as well as in the ventral striatum (e.g., Potvin et al., 2007; Beck et al., 2009; Barrós-Loscertales et al., 2011; Dombrovski et al., 2012; Koehler et al., 2015). In a twin-study, Ersche et al. (2011) found that the gray-matter volume of the basal ganglia is increased in drug addicts.

Similarly, resting state connectivity studies on impulsivity reached contrary results depending on whether implicit or explicit assessment of impulsivity was employed. Schmaal et al. (2012) measured impulsivity implicitly with a DD task and showed increased functional connectivity of the dorsal anterior cingulate cortex with midbrain regions, such as the ventral tegmental area and the substantia nigra, in individuals with high trait DD. In contrast, results from graph-theoretical analyses suggest that impulsivity (measured with the Barratt impulsivity scale) is associated with a disconnection between the prefrontal cortex and subcortical structures related to the reward system (Davis et al., 2013).

Increased activation has been related to increased gray matter, because of repeated allocations of several brain regions (Ilg et al., 2008). Gray matter changes in the striatum can be related to cell size changes of the neurons or glial cells, to the genesis of glial cells or neurons or to changes of the intracortical axonal architecture like synaptogenesis (May and Gaser, 2006). There is evidence for plasticity of the human brain to adapt to new situations (Ceccarelli et al., 2009). Ceccarelli et al. (2009) reported increased gray matter in the dorsomedial frontal cortex, the orbitofrontal cortex, and the precuneus for healthy adults after having a cognitive training.

Given the fact that the striatum and especially the caudate plays a major role in DD decisions (Hariri et al., 2006; Pine et al., 2009), an increased volume of the caudate and putamen for high impulsivity implicates that plasticity of gray-matter volume of relevant regions is required in high impulsivity to process time delayed reinforcements.

Differences between implicit and explicit assessment methods may also explain differences in behavioral results between the present and previous studies. Previously, using self-report measures, higher trait impulsivity has been reported in men compared to women (Chamorro et al., 2012) and higher age was related to improved self-control abilities and reduced impulsivity (Steinberg et al., 2008). In the present study, women and men displayed comparable values of the DD parameters *K* and *SbS*. Age was also not significantly correlated with *K* or *SbS* in the present results, in accordance with previous evidence (Smith and Hantula, 2008), despite the fact that questionnaire based impulsivity measures are correlated with age. Furthermore, Smith and Hantula (2008) also show that sex is consistently correlated with *K*, such that men devaluate rewards less than women. These findings are not consistent with the present study. Importantly, we found a positive correlation between *K* and gray-matter volume in the striatum whilst controlling for age and gender, suggesting that the relationship between DD and subcortical gray matter volumes was independent of these factors.

DD is highly correlated with trait impulsivity and can be treated as a stabile personality trait (Ohmura et al., 2006). There is evidence that several personality traits like extraversion or neuroticism are related to DD (Hirsh et al., 2008; Augustine and Larsen, 2011). Since personality traits as well as emotional processes can explain DD behavior (Hirsh et al., 2010), inconsistency in the functional as well as structural imaging literature might be better explained by considering this factors.

In conclusion, the current study provides strong evidence that impulsive decision-making is associated with increased gray-matter volume in subcortical structures within the mesolimbic reward system. Since impulsivity is a major vulnerability factor for several mental disorders (Bickel et al., 2012), these findings are of high clinical relevance.

## Conflict of Interest Statement

The authors declare that the research was conducted in the absence of any commercial or financial relationships that could be construed as a potential conflict of interest.

## Footnotes

^1^http://dbm.neuro.uni-jena.de/vbm/

^2^http://www.fil.ion.ucl.ac.uk/spm/

^3^http://surfer.nmr.mgh.harvard.edu/

^4^http://www.fmrib.ox.ac.uk/fsl
